# Adaptive control of ventilation using electrical stimulation in a biomechanical model

**DOI:** 10.1186/1471-2202-16-S1-P111

**Published:** 2015-12-18

**Authors:** Brian K Hillen, James J Abbas, Adeline Zbrzeski, Sylvie Renaud, Ranu Jung

**Affiliations:** 1 Department of Biomedical Engineering, Florida International University, Miami, FL, 33174, USA; 2 School of Biological and Health Systems Engineering, Arizona State University, Tempe, AZ, 85287, USA; 3 IMS Laboratory, CNRS UMR 5218,Institut Polytechnique de Bordeaux, Talence, 33405, France

## 

**Figure 1:**
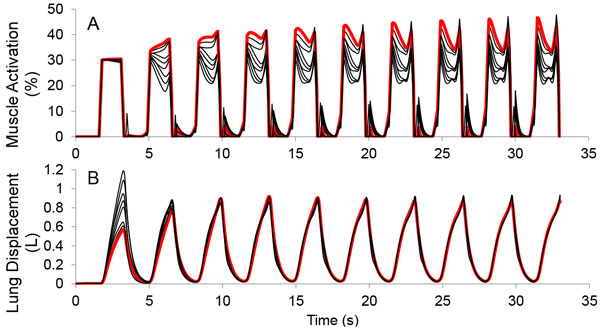
**A: Stimulation output for each biomechanical model tested for the first 10 breaths**. **B: **Lung volume trajectories for each biomechanical model. The trial on the system with the weakest muscle and greatest load (damping) is shown in red. Note that all models achieved the desired lung volume but required different stimulation patterns.

Cervical spinal cord injury (SCI) causes loss or impairment of control of respiratory muscles. Life-sustaining ventilation can be provided by mechanical ventilators (which have numerous side effects) or open-loop electrical stimulation respiratory pacing systems [1]. The use of adaptive control strategies in respiratory pacing systems can simplify initial setup procedures and allow the system to adjust stimulation values to account for changes due to muscle fatigue and/or respiratory demand. We have implemented a neural network based adaptive controller [2] with a biomechanical model of human ventilator dynamics and diaphragm stimulation in Simulink/SimMechanics/ Matlab. The adaptive controller uses sensor information to automatically determine a stimulation pattern that will produce a pre-specified desired lung volume trajectory. The controller uses a two-stage pattern generator/pattern shaper (PG/PS) structure which has successfully controlled leg movements in human subjects [3] and rats [4] using neuromuscular electrical stimulation. The biomechanical model incorporates a physiologically realistic Hill-type muscle model and a damped spring with non-linear compliance using published parameters for muscle geometry [5]. The parameters of the biomechanical model (muscle mass and lung damping) were varied +/-20% to simulate variation across a population to yield 9 sets of parameters. The quality of control and rate of adaptation achieved by the PG/PS controller were quantified by assessing the tracking error (difference between the actual and desired volume patterns) and the number of cycles needed to reach 5% error. Controller parameters were initialized to provide a nominal degree of ventilation during initial breaths. For each set of biomechanical parameters, the controller adapted stimulation values to achieve the same desired volume trajectory without any modification of initial controller values (Figure 1) and to achieve less than 5% error in 1-10 (mean 5.4 ± 2.6) cycles. This adaptive strategy will be investigated further in simulation as well as hardware implementations for testing in animal models.
